# Machine Learning Approach for Prescriptive Plant Breeding

**DOI:** 10.1038/s41598-019-53451-4

**Published:** 2019-11-20

**Authors:** Kyle A. Parmley, Race H. Higgins, Baskar Ganapathysubramanian, Soumik Sarkar, Asheesh K. Singh

**Affiliations:** 1 Department of Agronomy, Iowa State University, Ames, IA USA; 2 Department of Mechanical Engineering, Iowa State University, Ames, IA USA

**Keywords:** High-throughput screening, Plant breeding

## Abstract

We explored the capability of fusing high dimensional phenotypic trait (phenomic) data with a machine learning (ML) approach to provide plant breeders the tools to do both in-season seed yield (SY) prediction and prescriptive cultivar development for targeted agro-management practices (e.g., row spacing and seeding density). We phenotyped 32 SoyNAM parent genotypes in two independent studies each with contrasting agro-management treatments (two row spacing, three seeding densities). Phenotypic trait data (canopy temperature, chlorophyll content, hyperspectral reflectance, leaf area index, and light interception) were generated using an array of sensors at three growth stages during the growing season and seed yield (SY) determined by machine harvest. Random forest (RF) was used to train models for SY prediction using phenotypic traits (predictor variables) to identify the optimal temporal combination of variables to maximize accuracy and resource allocation. RF models were trained using data from both experiments and individually for each agro-management treatment. We report the most important traits agnostic of agro-management practices. Several predictor variables showed conditional importance dependent on the agro-management system. We assembled predictive models to enable in-season SY prediction, enabling the development of a framework to integrate phenomics information with powerful ML for prediction enabled prescriptive plant breeding.

## Introduction

Soybean [*Glycine max* (L.) Merr.] is the most economically important oilseed crop in the world because of its unique compositional profile as a dual source of high-quality oil and protein^1^. In 2017, land acreage dedicated to soybean production in the United States (US) reached record levels of 87 million acres^2^, due to a demand surge necessitating increased production. Globally, the US and Brazil are the two largest soybean producers while China, the primary consumer of soybean products, has steadily increased imports of soybean products^3^. The US soybean production area spans a vast and diverse geographic region from the southern US to the Northern Great Plains. Irrespective of region, the primary breeding objective is increasing seed yield (SY) of soybean to meet the needs of producers and other stakeholders.

Seed yield is a function of genetic potential, agronomic management, and environmental conditions that interact together, resulting in the observed SY phenotype. Improved agronomic practices and breeding efforts contribute a considerable proportion towards increased SY in soybean^4–6^. Breeders have made considerable improvement in the rate of genetic gain with estimates ranging from 22.6–43 kg ha^−1^ yr^−1^ ^4,5,7,8^. Concurrently, soybean producers have contributed by deploying diverse agronomic systems that balance their infrastructure set-up and production constraints (for example, abiotic and biotic stress) to achieve the maximum SY potential.

The diverse soybean agronomic systems that producers are currently deploying include different row spacing and seeding densities. The top two row spacing currently used in the US soybean production are 38 and 76 cm row-to-row distance, while varying other row spacing are also used^9^. The primary drivers in the surge in narrow row spacing (i.e., 38 cm) is to improve early season light interception^10,11^, decrease weed competition^12^, and thereby increase SY^13^; however, wider row spacing (i.e. 76 cm) also remains popular for better disease control^14^, and existing equipment infrastructure on some farms^13^. For seeding densities, producers consider optimizing the seeding rate to balance the economic and production systems, with an aim to maximize profitability. Modern soybean cultivars achieve maximum SY at high seeding densities but the rate of yield gain is curvilinear^6,15,16^. Reported benefits of high seeding densities are: decreased time to canopy coverage^6^, decreased weed competition^12^, and insurance to potential loss of stand during the growing season^17^. However, at lower seeding densities, modern soybean plants exhibit compensatory mechanisms through increased branching and SY on the branches^6^, therefore the decision of optimal seeding rate is not simple as very low seeding densities are unable to compensate for SY^6^. As producers adjust their production systems, the physiological mechanisms remain unclear precluding production specific cultivar development.

With the recent improvement of sensor and remote sensing technology, high throughput phenotyping (HTP) techniques are being deployed to quantify complex phenotypic traits, which in the past were either difficult to collect or required destructive sampling^18,19^. In soybean, studies have used HTP to identify several phenotypic traits (e.g., canopy coverage, canopy temperature, hyperspectral canopy reflectance, chlorophyll content) correlated with SY allowing breeders to investigate and identify important physiological mechanisms controlling the underlying genotypic variation in SY^7,20^. Studies have demonstrated the application of HTP in breeding activities to aide in cultivar selection using indirect selection techniques^21^, genome-wide association studies^22^, genomic selection^23,24^ and phenotypic prediction^25^. Modern aerial and ground-based HTP platforms provide a wide breadth of data to quantify the plant phenome across a spatial and temporal scale on thousands of genotypes generating a rich phenotypic trait data (phenomic) cube for breeding applications. To realize the value of phenomics assisted breeding, it is pertinent to leverage sensor technologies and advanced data analytics for prediction on each candidate cultivar across contrasting management systems.

The high dimensional phenotypic trait data cube generates copious amounts of data resulting in complex datasets. Traditional methods including regression-based techniques that are often limited in their ability to analyze high-dimensional data and are unable to capture complex and multivariate relationships between the predictor and response variables^26^. To effectively model these complex relationships without compromising on model interpretability, feature selection methods in machine learning (ML) minimize the number of predictors without compromising on model performance^27,28^. Additionally, ML approaches are equipped to exploit large datasets of contrasting data types making it a useful tool to leverage high dimensional phenotypic trait data in plant breeding efforts^29,30^.

The ML method ‘Random Forest’ (RF)^26^ has the ability to conduct regression, classification, and unsupervised learning and additionally estimate predictor importance for predictors given complex data, which is applicable to SY (response variable) prediction using phenotypic traits (predictor variables) in a spatio-temporal scale. Briefly, RF is an ensemble method that builds a collection of decision trees through the process of bagging, where a subset of the data is used for training of each decision tree and an additional level of randomness is included by randomly selecting, at each node, a subset of predictors. This approach ensures that the trees are de-correlated through integrating multiple rounds of randomness and increases prediction robustness by averaging the predictions across several weak learners. Additionally, another output of RF is an estimate of predictor importance for each predictor variable allowing the user to gain understanding of their relationship with the targeted trait (for example, SY). RF does not make any prior assumptions about the structure of the data capturing linear and non-linear dependencies between the predictor and the response variables, making it a suitable analytical tool for life sciences research and plant breeding applications. Among all feature selection algorithms, random forest (RF) has been deemed as one of the best prediction methods in a wide array of comparative studies^31–33^.

The aim of this study was to elucidate the relationship between SY and phenotypic traits under diverse agro-management systems for the development of predictive tools useful in prescriptive breeding efforts tailored to production systems. We evaluated 32 genotypes of the SoyNAM panel in two contrasting agro-management production systems (row spacing and seeding densities) across several environments. Phenotypic traits were collected during the soybean vegetative and reproductive growth stages using various remote sensors and end of season machine harvested SY. We identified the most important predictor variables for targeted agro-management systems using ML feature selection techniques and demonstrated their utility for a prescriptive plant breeding approach. We report that ML methods can be successfully used to identify agro-management specific predictors of seed yield. The integration of phenomics and ML will equip breeders with the analytical tools for optimized cultivar development and placement to targeted agro-management practices. This will enable an increase in the rate of genetic gain and stakeholder profitability, and improve research operational efficiency.

## Materials and Methods

### Germplasm and treatment design

A subset (n = 32) of the soybean nested association mapping (SoyNAM) parents (Supplementary Table S1) adapted to the maturity requirements of central Iowa, USA, were evaluated across nine location environments^34^. These genotypes represent the broader soybean germplasm based on their SY, diverse ancestry, or stress tolerance while representing a broad genetic diversity of soybean. Two independent but related projects were undertaken: (1) Treatments of row spacing (IA-RS), and (2) Treatments of seeding densities (IA-SD). In IA-RS tests, we measured genotype performance of 32 genotypes in 38 and 76 cm row spacing treatments seeded at 345,000 seeds ha^−1^. IA-RS tests were grown at five environments (in 2015: Boone and Story county, IA; in 2016: Boone county, IA and Cass county, IA). In IA-SD tests, all treatments grown in 76 cm row spacing, genotypes were evaluated in three seeding density treatments of low = 124,000 plants ha^−1^, medium (commercial density) = 346,000 plants ha^−1^, and high = 568,000 plants ha^−1^. IA-SD was grown at four environments (in 2014: story county, IA; in 2015: Story, Boone, and Warren county, IA). See Supplementary Table S2 for additional information on location GPS coordinates and observed environmental conditions.

The experimental design was a randomized complete block (RCB) design with three replications per genotype and treatment level. Plots were seeded with an Almaco cone planter (Almaco, Nevada, IA) with row units on 76 cm row spacing. Except IA-RS 38 cm treatment, all other experimental units consisted of four rows for all treatments, 4.57 m plot length with 0.91 m alley between plots, and 76 cm row to row distance. Plots with 38 cm row spacing consisted of eight rows and were established in the following manner: after an initial pass of seeding plots on 76 cm spacing, the GPS enabled planter (SkyTrip-Almaco, Nevada, IA) was then re-positioned to the front of the field and a second pass made over the field by positioning the new rows 38 cm left from the initial pass. In the second pass, row cones were filled with autoclaved (dead) soybean seed. There was no planter tire traffic on the harvested middle two rows (76 cm row to row spacing plots) and harvested middle four rows (38 cm row to row spacing treatment plots). Visual observation was done to ensure that none of the autoclaved seed emerged in the non-38 cm treatments and stand counts taken in all experiments to ensure that planter traffic had no impact on row spacing treatments and that the targeted seeding densities were achieved (data not shown). To reduce the impact of edge effects in both tests, a soybean cultivar was planted around all sides of the field as border plots. At physiological maturity (R8), middle rows of each plot were harvested with an Almaco SPC20 combine equipped with a platform header. Seed yield was adjusted to 13% moisture and SY in kg ha^−1^ recorded.

### Phenotypic traits

Plots were phenotyped f at three growth stages; S1: late vegetative to early flowering (V5-R1), S2: full flowering to early pod set (R2–3), S3: Seed development and fill (R5–6)^35^. These growth stages were targeted due to their biological significance and previous research in soybean confirming the prediction importance to SY^4,20,36,37^. All trait data was collected from the middle two (all non-38 cm spacing plots) or four rows (all 38 cm row spacing plots) from all plots while remaining at least 1.0 m from the plot edge to minimize the impact of the alley on trait performance.

Leaf chlorophyll content (SPAD) was measured using the SPAD index with a Minolta SPAD-502 chlorophyll meter (Konica Minolta Optics, Inc., Japan), which measures absorbance at 650 and 940 nm. Measurements were collected from 10 fully developed leaves in the upper portion of the canopy and values averaged for each plot.

Canopy temperature (CT) was measured with Apogee SI-111 infrared radiometers (Apogee Instruments Inc., USA). Measurements was collected between the hours of 1200 to 1500 h on sunny cloudless days when wind was <2.24 m s^−1^. The sensor was placed at a height of 0.3 m above the canopy and positioned at 65° below horizontal. To minimize the impact of environmental conditions on genotype performance and for time sensitivity, separate SI-111 infrared radiometers were used to collect data in each replication. Four measurements were collected from random locations within the middle rows of the plot and an average value was computed.

Leaf area index (LAI) and leaf mean tilt angle (MTA) were measured using a LAI-2200C plant canopy analyzer (Li-Cor, Inc., USA), which measures transmitted light at five angles using a fish-eye optical sensor. LAI was determined along a diagonal transect between the middle rows using a 45° view cap. To account for temporal variation in light quality, a scattering correction measurement was recorded between every two ranges of plots (~10 min), this process was repeated until completion of data collection. The scattering correction was then applied to every plot to adjust values to temporal fluctuations in light quality. Measurements consisted of one above-canopy measurement followed by six and four evenly spaced below-canopy measurements for S1 and remaining growth stages, respectively, due to a smaller canopy size at S1 and a need to reduce measurement error.

Intercepted photosynthetically active radiation (iPAR) was measured between the hours of 1100 to 1500 h on sunny cloudless days with LI-191R line quantum sensor (Li-Cor, Inc., USA), which measures light from 400 to 700 nm along a one-meter rod. In the 2014 and 2015 growing seasons, one sensor was used for collection of iPAR. iPAR was determined by first measuring PAR light intensity above the canopy (*P*_*a*_) then collecting PAR transmitted light quality below the canopy (*P*_*b*_), at the soil surface, by placing the sensor perpendicular to the middle rows. In 2016, with the availability of an extra sensor, a small modification was made where two sensors were simultaneously used: one above the canopy and the second on the soil surface perpendicular to the middle rows. This provided a gain in efficiency of data collection in a more time sensitive manner. Light interception was calculated as:

$$iPAR=1-(\frac{{P}_{a}}{{P}_{b}})\cdot $$

Canopy spectral reflectance was measured using a FieldSpec® 4 Hi-Res (ASD Inc., USA) spectroradiometer, which measures reflected electromagnetic energy from 350 to 2500 nm with a 1 nm interval. Reflectance measurement were recorded on cloudless days between ±2 h of solar noon and calibrated every 20 minutes during data collection by normalizing to a white reference panel (Specralon®, Labsphere Inc., USA). Canopy reflectance was collected by positioning the fiber optic cable 1.0 m above the canopy in the nadir position. Two scans were collected directly over each row and reflectance values averaged. Vegetative indices (VI) were calculated from the average reflectance values for each plot (Supplementary Table S3).

### Data processing and analysis

In total, we recorded 2250 instances of data from all locations and experiments (combination of genotype, treatments of RS and SD, replications, environments, response and predictor traits). The dataset was composed of 22 traits for each of the three growth stages. We utilize trait abbreviation followed by the growth stage at which data collection was made (e.g., LAI_S1 represent Leaf area index (LAI), and S1 represents the growth stage). For VI, we used “VI” followed by the stage and the abbreviation of the VI index (e.g., VI_S1_NDVI).

To determine the effect of management treatments (RS and SD) on SY and simultaneously conduct outlier analysis on all trait observations, the following mixed model was fit to the RCB design:

$${y}_{ijkl}=\mu +{l}_{i}+{g}_{j}+g{l}_{ij}+{t}_{k}+g{t}_{jk}+{r}_{l(i)}+{\in }_{ijkl}$$

Where *y*_*ijkl*_ is the phenotypic observation for the trait of interest, *μ* is the overall mean, *l*_*i*_ is the fixed effect of location (environment) *i*, *g*_*j*_ is the fixed genotype effect of entry *j*, *gl*_*ij*_ is the interaction effect between genotype *i* and location *j*, *t*_*k*_ is the fixed effect of the management treatment (RS and SD) *k*, *gt*_*jk*_ is the interaction effect of genotype *i* and management treatment *k*, *r*_*k*(*i*)_ is the random effect of the *l* replication nested within location distributed as iid ${r}_{l(i)} \sim N(0,{\sigma }_{l}^{2})$, and ∈_*ijkl*_ is the residual error variance distributed as iid. To identify inconsistencies in the data, outliers were removed by calculating studentized residuals for each observation of each trait and outliers excluded from the analysis with values ±3^38^. Variance components were calculated for each management system from the mixed linear model by substituting the fixed effect terms for random effects and removing the management term and its interaction from the mixed linear model. Broad sense heritability (H^2^) for SY was computed as the ratio of genetic variance to phenotypic variance on an entry-mean basis according to^39^:

$${H}^{2}=\frac{{\sigma }_{g}^{2}}{{\sigma }_{g}^{2}+\frac{{\sigma }_{gl}^{2}}{r}+\frac{{\sigma }_{\in }^{2}}{rl}}$$

where ${\sigma }_{g}^{2}$ is the genotypic variance, ${\sigma }_{gl}^{2}$ is the variance of the genotype by environment interaction, and ${\sigma }_{\in }^{2}$ is the residual error variance. The number of replications and environments are represented by *r* and *l*, respectively. The lme4 package in R v3.3.3^40^ was used to fit linear models, conduct outlier analysis, and to compute variance components.

We attempted to assemble a complete dataset with phenotypic observations from all location, growth stages, and traits but weather and logistical constraints prohibited such a complete dataset. The missForest package^41^ in R was used for imputation of missing predictor traits. The missForest package allowed imputation of categorical and continuous multivariate data by using an iterative RF imputation scheme. The missforest package is considered a suitable method for such scenarios, i.e., missing or incomplete dataset^41–43^.

Genotype best linear unbiased predictors (BLUPs) were calculated for all predictor and response variables by fitting the following mixed model to the RCB design for each management system-genotype combination at each location:

$${y}_{ij}=\mu +{r}_{i}+{g}_{j}+{\in }_{ij}$$

where *y*_*ij*_ is the observed phenotype, *μ* is the overall mean, *r*_*i*_ is the random effect for the *i* replication distributed as iid ${r}_{i} \sim N(0,{\sigma }_{i}^{2})$, *g*_*j*_ is the random effect for genotype *j* distributed as iid ${g}_{j} \sim N(0,{\sigma }_{j}^{2})$, and ∈_*ijkl*_ is the residual error variance distributed as iid. These data were then used for model training and prediction.

### Random forest model training

In this study, we utilized RF for prediction of SY (response) using phenotypic traits as predictor variables. Prior to model training, we removed observations with missing yield data and predictor traits were normalized using z-score normalization:

$$z=\frac{x-\overline{x}}{\sigma }$$

where *x* is the observed trait value, $\overline{x}$ is the mean trait value, and *σ* is the standard deviation of the trait. Normalization step was used so that all predictor traits have similar magnitude variability, and thus impact the model in consistent ways. During model training the data was partitioned in training (80%) and testing (20%) datasets and model trained using 10-fold cross validation to gauge training performance, while the testing set was used for model validation consisting of independent data not used to train the model. Model performance was assessed by computing the coefficient of determination (R^2^) and root mean square error (RMSE) for both the out-of-bag (OOB) training and test data. To accurately estimate model performance and stability of the model, we repeated the training and testing procedure ten times and averaged performance metrics across iterations. We report model predictive ability as the average R^2^ of regression models and average of several classification metrics for classifier models. Model training, hyperparameter optimization, and testing was carried out with the randomForest^44^ and caret^45^ packages implemented in R.

To demonstrate the utility of RF for phenomics-assisted prescriptive breeding, we present result from a four-tiered approach demonstrating our proposed framework that will enable prescriptive cultivar development:

### Predictor importance

Using phenotypic traits (predictors), we trained a RF model using data fused from both experiments (combined data from IA-SD and IA-RS) and then subsequently learned models for each of the individual treatment levels in both studies. During model training we computed feature attribution for predictor traits using the ‘varImp’ function to investigate predictor variables relationship with SY enabling the identification of spatio-temporal physiological and canopy trait dependencies on SY. The ‘varImp’ function computes feature importance by randomly permuting trait data and recording the impact on model accuracy on tree-by-tree basis and computing the mean impact across all trees. Importance parameters were computed for all ten iterations and mean reported for each predictor trait.

### Recursive feature elimination

To decrease model complexity and validate predictor importance analysis, we performed a recursive feature elimination (RFE) selection. RFE is a backward elimination procedure for all management treatment levels. It combined datasets and subsequently identified predictor variables selected in five or more of the ten training-testing iterations. Conversely, while the optimal model is most desirable, we also investigated minimizing the number of predictors in the final model to minimize the cost and time associated with data collection. Hence, we utilized the ‘pickSizeTolerance’ function in the caret package that identifies an acceptable model with fewer predictors by allowing higher error while falling within a user defined threshold range of the optimal model. The ‘pickSizeTolerance’ function selects a model containing fewer predictor traits within the bounds of a user-defined threshold metric. We utilized out-of-box (OOB) RMSE and a 5% tolerance threshold to identify models with acceptable performance but with fewer predictors. Once a subset of predictors was identified, we re-trained the models and measured predictive ability and contrasted that with the model that included all predictors in the respective management systems. In addition to the evaluation of predictive ability by comparing predicted to actual values we chose a hypothetical 20% selection intensity, which bears similarity to a practical breeding programs’ early generation testing and selection to determine breeding selection accuracy (defined as proportion of correct decisions divided by the total number of decision). Models selected with only the most important predictor traits were used to make predictions on the test dataset and performance assessed using a select subset of metrics that are applicable to plant breeding operations: balanced accuracy (BACC), precision (PRE), sensitivity (SEN), and specificity (SPE) according to the confusion matrix which classifies selection decisions as true positive (TP), true negative (TN), false positive (FP), and false negative (FN) (Supplementary Fig. S1). With these performance parameters we can measure the performance of a model for use in early generation plant breeding selections.

### Genotype agro-management fit and phenomics-enabled prediction

To identify genotypes with specific agro-management adaptations, we computed genotypic SY BLUP deviation (*D*_*g*_) between 38 cm and 76 cm row-spacing for IA-SD study and medium and low seeding density for IA-SD. We then determined genotype specific agro-management system adaptation (“fit”) by computing the mean genotypic deviation (*μ*_*g*_) of each study to assign genotypes to classes according to each genotypes response in contrasting management systems facilitating informative comparisons.

IA-RS experiments: >μ_g_ ± 1 St. Dev. [Narrow row-spacing (N)]; ≸ μ_g_ ± 1 St. Dev. [Universal (U)]; <μ_g_ ± 1 St. Dev. [Wide row-spacing (W)]

IA-SD experiments: >μ_g_ ± 1 St. Dev. [Medium seeding density (M)]; ≸ μ_g_ ± 1 St. Dev. [Universal (U)], <μ_g_ ± 1 St. Dev. [Low seeding density (L)

Using genotypes fit as a response, we fit a RF classifier for both studies using the subset of predictor traits from both management systems and computed model predictive ability. To deal with class imbalance we utilized the ‘upSample’ function and model performance metrics were generated for each class in both scenarios and results averaged over the ten iterations. The ‘upSample’ function deals with classification problems in the case of frequency disparities between classes by randomly sampling the minority class to the same size as the majority class.

### Breeding program design to maximize prescriptive breeding efforts

To evaluate the effect of the agro-management system utilized in the breeding process on predicting genotype management fit, we trained RF classifiers using data from each treatment levels to predict genotype management fit. We then compared model classification performance metrics between management systems in their respective studies to determine management systems that maximize the identification genotype management fit.

## Results

### Genotype performance

The SY performance of the 32 SoyNAM genotypes in this study was measured in contrasting agro-management systems in two independent studies (IA-RS and IA-SD). A significant effect of both genotype and agro-management treatments was detected in both studies (Supplementary Table S4). We observed higher mean SY in IA-RS when compared to IA-SD and contrastingly higher SY variation in IA-SD (Supplementary Table S5). Heritability (H^2^) estimates for SY on an entry mean basis ranged from 0.78–0.96 with lower values in IA-SD study (Supplementary Table S5). In the IA-RS experiment, narrow rows increased SY on average by 56.8 kg ha^−1^ but no clear pattern was observed among genotypes in varying row spacing. When comparing genotype SY performance in row spacing treatments, 69% had superior SY response in 38 cm, while 31% had higher SY in 76 cm row spacing. Genotypes with narrow row preference produced on average 125 kg ha^−1^ higher SY when compared to their performance in wide rows, while cultivars with preference to wide rows yielded 94 kg ha^−1^ on average higher SY than when grown in 38 cm row spacing. We observed a wide range in SY of genotypes with favorable response to narrow row ranging from 1–236 kg ha^−1^ while PI 518751 exhibited the largest response. The range in SY response to 76 cm versus 38 cm row spacing ranged from 32–154 kg ha^−1^ with LG94–1128 exhibiting the largest response. In IA-SD, a curvilinear response between SY and planting density was observed with maximum yields achieved in high seeding density while not significantly different from med density treatment (Supplementary Table S4). Sixteen (50%) genotypes achieved maximum SY when grown in med seeding density while all but two (LG05–4832 and Prohio) yielded highest when grown at the highest seeding density. When comparing genotype performance when grown in low to med seeding density SY decreased on average 10% but some genotypes were more negatively impacted (e.g., NE3001: 29% reduction) while other genotypes (e.g., 5M20-2-5-2: 4.4% reduction) had compensatory SY ability.

### Predictor importance in predicting sy in varying agro-management systems

**Figure 1:**
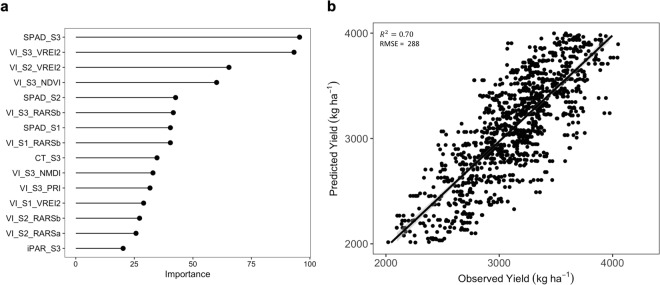
(**a**) Predictor importance and (**b**) model performance of a Random Forest model trained using data from the row spacing and seeding density studies and from all treatments for seed yield prediction.

The major predictor variables to predict SY were identified in the combined (IA-RS, IA-SD) analyses, and included: SPAD_S3, VI_S3_VREI2, VI_S2_VREI2, VI_S3_NDVI, and SPAD_S2 as the top five most important variables (Fig. 1a). Outside of the top five but consistent with the above traits, SPAD_S1 and VI_S1_VREI2 were identified to be in the top 15 most important. Interestingly, VI_RARSb was identified in the top 15 predictors at all three growth stages. In summary, ten VI, three SPAD, one CT, and one iPAR were among the top 15 predictor variables.

Among these, 8/15 were from S3, 4/15 from S2, and 3/15 from S1 were included.

**Figure 2:**
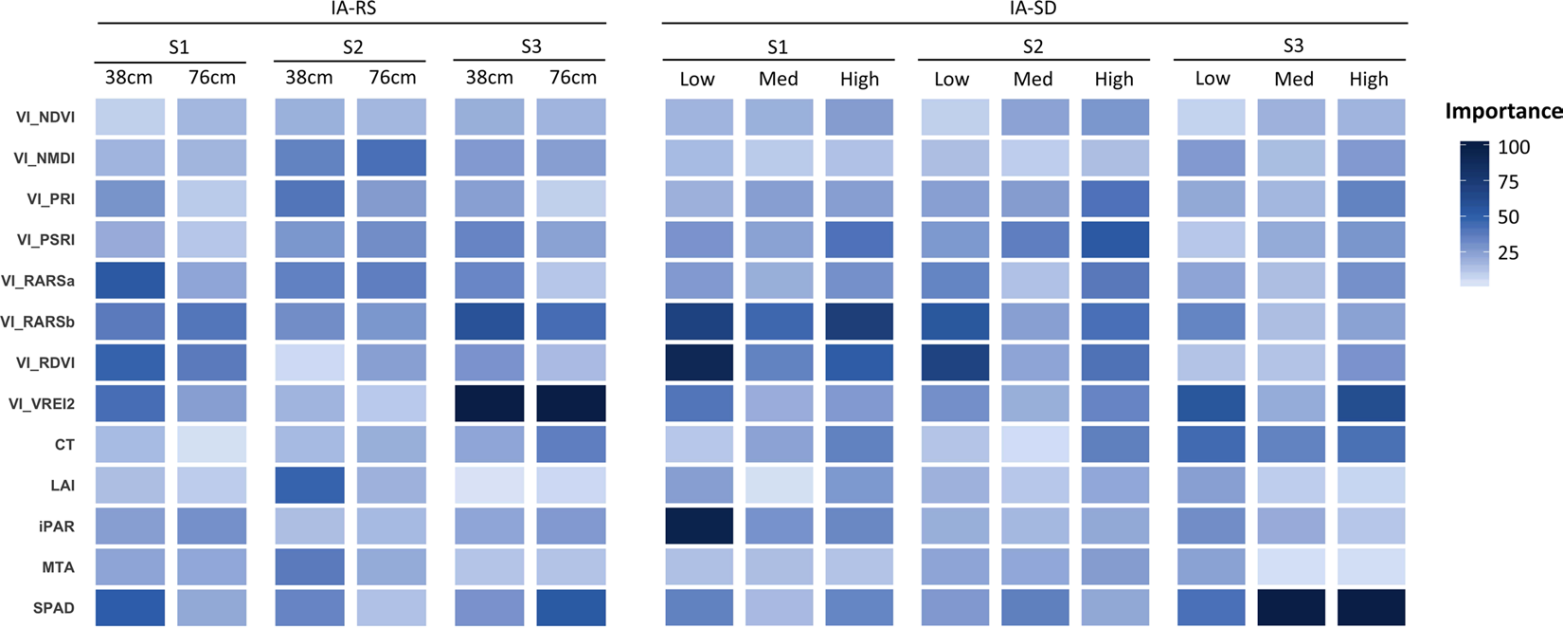
Heatmap of predictor feature importance computed for each agro-management treatment level from the row spacing and seeding density studies. Darker blue colors indicate higher importance of traits for seed yield prediction.

Using the RF model constructed with all predictor variables, an average predictive ability of 70% was observed for SY prediction across all test sets (Fig. 1b). Along with training a RF model with the combined dataset, we parameterized models for each agro-management treatment level in each of the two treatment experiments, IA-RS and IA-SD. Mean predictive ability for all treatments was 66% while IA-RS ranged from 50–53% and for IA-SD ranged from 69–82% (Table 1). The Fig. 2 provides a visual map of the RF model based predictor importance for all measured traits, including the 15 most important variables shown in Fig. 1. Based on the importance map, which reflects the most important predictor variable in dark blue, best prediction is possible at the S3 stage; however, several variables are important at all stages including VI_VREI2, VI_RARSb, iPAR, and SPAD.

**Table 1:** Model performance of Random Forest models trained using observations from each agro-management treatment from row spacing and seeding density studies.

Study	Treatment	OOB Training		Testing	
		RMSE	R^2^	RMSE	R^2^
IA-RS	38 cm	328.2	0.57	222.6	0.53
	76 cm	334.8	0.50	204.9	0.50
IA-SD	Low (124,000 plants/ha)	232.7	0.83	185.3	0.82
	Med (346,000 plants/ha)	303.9	0.71	241.1	0.69
	High (568,000 plants/ha)	299.7	0.71	209.4	0.74

OOB = Out of Bag, RMSE = Root Mean Square Error. IA-RS = row spacing study. IA-SD = seeding density study.

No clear trend was observed among predictor variables differing between the two row spacing treatments. Using predictor importance estimates and a deviation of importance at 25, VI_RARSa_S1 and LAI_S2 were different between the 38 cm and 76 cm row spacings. However, a trend was observed among SPAD_S1 and S2, VI_PRI_S2 while not higher than our deviation of importance criteria but had a notable difference between the two row spacings. In the IA-SD experiments, SPAD_S3 was the best predictor for high and medium densities while iPAR_S1 was most important for low density. The iPAR_S1, VI_VREI2_S1, and SPAD_S3 at low density was different from both high and medium density for the respective predictors, and VI_RARSb_S1 and VI_VREI2_S3 at high density was different between the high and medium densities. VI_RDVI_S2 was different between low and medium densities.

### Recursive feature elimination (RFE) model performance

**Figure 3:**
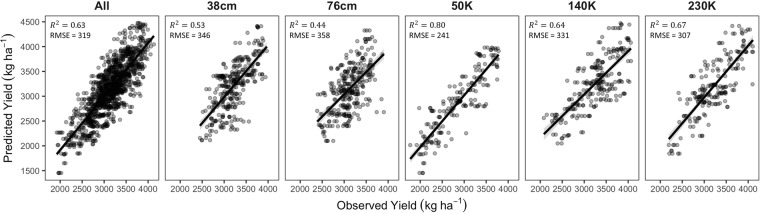
Random Forest model performance for seed yield prediction after the Recursive Feature Elimination process to remove uninformative predictors and to minimize data collection efforts. Model performance was accessed on an independent testing set not used during model training.

Further validation and data dimensionality reduction was provided through RFE. From the full list of all predictor variables (22 traits per growth stage for a total of 66 variables), ML enabled analyses identified the 15 most informative traits for SY prediction with only a slight loss in predictive ability or increase in error (Fig. 3; Supplementary Table S6). RFE results confirmed the earliest observations of the most important variables (Supplementary Table S7). After the initial RFE analysis, an average of 23 predictors was selected and for further reduction in model complexity we selected models with a threshold of 5% greater RMSE reducing the average number of predictors per management system to six. The number of features included in these reduced models ranged from eight to three predictors, reducing the dimension of the dataset even further resulting in a near ten-fold reduction in the total number of predictors per model. To evaluate performance of the RF models using only a small subset of the predictors, we projected predictions onto the test data set which was excluded during model training for model validation and compared the difference between predictive ability of models with a subset to the congruent model trained using all features. Higher predictive ability was observed for each of the three IA-SD treatments compared to the two IA-RS treatments (Fig. 3). We observed higher RMSE for all models trained with fewer predictors and only a slight reduction in predictive ability (0.07 for combined data, 0.09 for 38 cm, 0.10 for 76 cm, 0.02 for low seeding density, 0.05 for medium seeding density, and 0.07 for high seeding density). Subsequently, we gauged model performance to rank genotype SY performance using RF models following the RFE process.

We observed moderate to high BACC and SEN values ranging from 0.72–0.88 and 0.55–0.81, respectively. Higher SEN values were observed in IA-SD with average value of 0.73 compared to 0.55 in IA-RS. High estimates for SPE were observed, ranging from 0.91–0.95.

### Genotype agro-management fit and phenomics-enabled prediction

We observed a mean deviation of 59 ± 115 kg ha^−1^ and 332 ± 178 kg ha^−1^ for IA-RS and IA-SD, respectively (Supplementary Fig. S2). Five genotypes were identified with adaptation to narrow row spacing with a mean deviation of 218 kg ha^−1^ and eight genotypes with adaptation to wide row spacing with a mean deviation of −98 kg ha^−1^. Six genotypes were classified as adapted to low seeding density and five adapted to medium seeding density with mean deviation of 70 kg ha^−1^ and 600 kg ha^−1^, respectively. No pattern in agro-management fit of genotypes across the two studies was observed.

Across both experiments, we noticed a consistent trend among classification metrics where SPE and BACC was highest while other metrics trended lower (Supplementary Fig. S3). The RF classifier in IA-RS had high SPE ranging from 0.75–0.85 in the wide and narrow row spacing adaptation classes while a moderate value (0.47) for unresponsive adaptation class. Conversely SEN and PRE demonstrated an opposite pattern to that of SPE for the narrow and wide row spacing classes, while predictive ability of the unresponsive cohort remained consistent at moderate levels. For IA-SD we observed a similar trend in predictive ability of the RF model for performance metrics with high SPE ranging from 0.89–0.91 for low and medium seeding density adaptation classes and a moderate value (0.21) for the unresponsive class. Excluding the unresponsive cohort from both studies, we observed a similar trend among predictive ability in IA-RS and IA-SD for SEN. BACC remained consistent across the three adaptation classes in both studies with average values of 0.52 and 0.51 for IA-RS and IA-SD, respectively.

### Breeding program design to optimize prescriptive breeding efforts

Across all management treatment levels, predictive ability followed a similar trend among performance metrics when training data was used from all treatments compared to when it was limited to just one (Supplementary Fig. S3). Similarly, for most metrics model performance seemed to vary only slightly dependent on the management treatment level the model was trained with. For example, we observed high SPE of 0.90 and 0.69 for narrow and wide row spacing adaptation, respectively, when training data was derived from plots with narrow rows; while high SPE of 0.83 and 0.73 was observed when training data was sourced from wide rows. This same pattern was observed in performance metrics in IA-SD when comparing the source of the training data.

While most parameters remained stable regardless of the source of training data, for wide row spacing fit cohort in IA-RS, SPE of 0.75 was observed when all data was included in training and 0.22 and 0.28 for narrow and wide row spacing, respectively. Dissimilar to this finding in the unadapted cohort, SPE was greatest when training data was included from individual treatment with the largest value of 0.73 from wide row spacing. Furthermore, we observed a similar trend for SEN of 0.65 when all data was included in model training and 0.06 and 0.11 for narrow and wide row spacing, respectively.

In IA-SD we observed consistent performance among classification metrics with only slight variation when training data was sourced from contrasting agro-management systems. These results suggest that breeding programs can leverage one pipeline to optimize cultivar fit to several producer agro-management systems thereby decreasing operational costs incurred by such programs.

## Discussion

We propose a methdology that leverages important phenotypic predictors of contrasting treatment levels for genotype adaptation fit classes prediction to explore the capability of deploying prescriptive breeding techniques for data driven product placement from one breeding pipeline. The lack of information on deploying traits for predicting yield on a continual time series basis precludes physio-genetic approaches to improve breeding efficiency; however, with the recent advances in higher throughput phenomics enabling technologies and associated data analytics presents exciting opportunities for plant breeders and scientists to further prescriptive cultivar development. To achieve the overarching goal of developing tools for prescriptive cultivar development using phenomics assisted breeding techniques, we conducted a large scale agronomic-physiological-genetic trial. We observed improved SY performance in narrow rows compared to wider rows^10,46,47^, and a lack of SY gain in extremely higher planting densities^6,16^. Interestingly, our results suggest these genotypes may have potential preferences to agro-management systems (e.g., higher yield in 38 cm compared to 76 cm row spacing), which is an exciting finding for production agriculture and presents opportunities for prescriptive breeding. Using modern analytical techniques to identify phenotypic predictors of SY, we leveraged this knowledge to deploy predictive models for both SY and agro-management fit prediction. More work is needed to characterize the extent of phenotypic plasticity in SY response to contrasting agro-management systems and necessitates future studies to unravel the genetic and physio-morphological underpinnings of these responses.

Due to the large suite of phenotypic traits measured over time series (phenomics), we were able to concurrently assess the importance of each variable in SY prediction across differing management practices on a time series scale. This was motivated with existing knowledge that genotypes utilize varying traits to achieve the final yield^20,48,49^, empowering breeders to manipulate these traits to increase SY^50^. Such studies need the usage of ML algorithms that are more suited to complex biological problems due to a lack of statistical assumptions on trait dependencies and relationships. To identify important predictor traits that are driving the SY response, a ML algorithm specifically RF was used to overcome one of the inherent lacunae of most ML tools, i.e., the lack of interpretability. The additional motivation for using ML algorithms was to explore the relationships between predictors and response variables to reveal important biological relationships among response and predictor variables, i.e., determine the effect of a predictor traits on the response variable (SY) while marginalizing all other informative predictors referred to as partial dependence plots^51^. With high dimensional data cubes that can be assembled with modern sensing technology, breeders are able to identify the best predictor variables and determine the biological relationship across numerous agro-management treatments.

Later growth stage time points were more predictive of SY irrespective of row spacing or planting density treatments and are consistent with previous studies made in soybean^36,37^ as well as those made in other crop species^52,53^. Generally, later growth stages may be more influential in determining final SY^36^ but there has been little empirical evidence of predictor importance for prescriptive cultivar development to validate this assumption. Across different row spacing and planting density treatments, the foremost predictors of SY were identified at S2 and S3 growth stages and relate to chlorophyll concentration (for example, SPAD, VI_VREI2) and the water status of the canopy (for example, CT), which are important contributors to the US historical genetic gain of soybean^7,20,54^. Increased chlorophyll has been linked to greater photosynthetic capacity in modern cultivars^55^, and the negative association between CT reduction and SY increase has been established^20,56,57^. For VI_VREI2, regardless of the agro-management treatment and growth stage, an inverse relationship between SY and VI_VREI2 was observed and supports similar studies in non-soybean crops on the negative relationship between VI_VREI2 values and chlorophyll content^58^.

Interestingly, LAI was an important predictor trait for both 38 cm at the S2 growth stage and high seeding density at the S1 growth stage which seems to support earlier studies on the importance of optimal LAI values when management systems are utilized for rapid canopy closure^59,60^. This result illustrate the interaction between increased early season canopy size and SY and demonstrate the importance of maximizing early season light interception while minimizing plant size during the reproductive growth stages to channel more available energy towards seed production^59^. The importance of multiple indices (VI_NMDI, VI_RARSa, and VI_RARSb are used for approximating water content, chlorophyll a, and chlorophyll b, respectively) for predicting SY indicate the complexity of SY and its prediction.

In addition to the main predictors, the next steps were to develop the best SY prediction using the suite of predictor traits. The prediction-models and visualization approaches revealed that a cocktail of predictors are needed to achieve SY prediction which may be reflective of the underlying complexity of traits responsible (Fig. 2). Furthermore, the predictor variables identified with contrasting levels of importance conditional on the agro-management system indicate that genotypes are deploying different physio-morphological mechanisms to achieve final SY. This finding suggests that breeders should identify and leverage predictor variables with contrasting importance to deliver cultivars with an optimized yield package in prescriptive breeding efforts. In general, we observed a wide distribution in importance of traits with very few (VI_VREI2, VI_RARSa, and SPAD) having consistent high importance regardless of management and growth stage. These observations emphasize the need for cost effective and user-friendly sensors and platforms with enough resolution and throughput during the crop season. These results are useful as any phenomics enabled selection strategy depends on time and cost constraints. Since we report results from three growth stages, it is unclear (but likely) that additional of more data collection dates will provide further insights on the predictor variables.

RFE was an effective analytic tool to identify an informative subset of predictors that optimizes prediction power, and reported the most important variables across and within each agro-management treatment to facilitate iterative approaches to work with a smaller set of trait collection^31,32,61^. These general and treatment specific trends between predictor and response variables provide directional modification strategies for use in plant breeding techniques to improve SY using multi-trait phenotypic selection^62^ or multi-trait genomic selection^63^ schemes.

As we expected, RFE confirmed the predictor importance analysis by selecting the predictors with highest importance in each agro-management system. The resulting models were established with dissimilar predictor variables and temporal growth stages from which to collect the data further strengthening the argument in support of genotypes deploying complex mechanism to achieve SY. While yield prediction may be important for producers to access productivity, breeders are faced with a binary task (select for or against) when making advancement decisions and often have little data during early generation testing^64^. Therefore, phenomics data, standalone or in conjunction with genomic data, have the ability to enable better selection decisions^63^.

To determine the applicability of phenomics enabled selection, we selected classification performance metrics applicable to breeding program efficiencies to access model performance; wherein moderate too high BACC levels indicated that prediction models correctly ranked plot level SY when comparing to observed values. Additionally, models had high SPE indicating that candidate cultivars with low yield can effectively be selected against providing increased confidence to the breeder. RF models had moderate SEN indicating that high yielding cultivars can correctly be identified. Importantly, this approach would equip breeders with the ability to make in-season yield prediction of candidate genotypes and subsequently more phenotyping resources can be optimally directed to complement remote sensing techniques, as well as provide selection guidelines in case of a non-normal or catastrophic natural event damaging selection site(s).

Elucidation of genotypes with synergistic adaptation to unique agro-management systems will permit breeders to deliver customized cultivars to bolster on-farm profitability by harnessing the synergistic G x M effect. However, this has eluded breeders as they are often limited by the scale of their testing footprint to test and make cultivar-management recommendations. With modern big-data analytic capabilities, breeders are now equipped with the tools to unravel key phenomic and genomic predictors driving the measured response in contrasting management systems.

To demonstrate a potential framework of prescriptive breeding, we first identified cohorts of genotypes with differential response to row spacing and seeding density treatments. Surprisingly, we observed a consistent trend in both studies where genotypes varied in their SY response to contrasting treatment levels. While more work is needed to corroborate these findings, this suggests the need for prescriptive breeding strategies to exploit genotype-management synergies^65^. Thus, we have devised the following strategy to leverage high dimension phenomic trait data for prescriptive breeding.

We did not observe a pattern among genetic background and management adaptation demonstrating that modern cultivars have not been optimized to certain management systems. This finding of differential SY performance dependent upon the agro-management system is consistent with other crop species^66,67^ suggesting a need for prescriptive breeding solutions. According to a USDA survey, soybean producers use three row spacing’s treatments for more than 90% of planted acreage in the US making such prescriptive breeding methodologies important to breeding programs to produce more competitive cultivars for different management systems.

Model performance parameters indicated high SPE and BACC indicating that ML tools such as RF can be deployed with high confidence to correctly discard, an important step in plant breeding during early generation testing^68^.While improving detection power of TP incidences warrants additional work, these results are evidence of the utility of RF as a prediction tool, consistent with other researchers findings^31,69^.

While this research required intensive ground-based data collection, these sensors have the ability to be deployed at scale on ground rovers and un-manned aerial vehicles for high-throughput phenotyping efforts. Therefore, phenomics-assisted breeding has the potential to revolutionize breeding program design by allowing breeders to understand complex trait relationships while integrating stakeholder agro-management information to drive breeding objectives^18^. However, operating breeding pipelines with multiple objectives is expensive and resource intensive necessitating that a singular breeding pipeline be utilized to limit operational costs and combine these in an optimized genomic breeding strategy^70^.

Overall, we found that ML has the potential to enable genotype management fit prediction regardless of the source of training data used. Our work demonstrates how modern plant breeding programs can leverage spatio-temporal phenotypic trait information and modern analytical tools to optimize the cultivar development and placement process, and continual additional work in this research area is needed. Determining cultivar management fit before entering resource intensive testing will allow for more efficient use of resources by only testing candidate cultivars in their targeted systems.

## Conclusions

The detection of: (a) universally important phenotypic traits across row spacing and planting density treatments, and (b) treatment specific predictor traits important for SY prediction, indicate the necessity to determine the complement of useful and predictive traits. These will assist in breeding selections as well as better elucidate SY mechanisms on a spatio-temporal scale.

One of the ongoing interests in plant breeding programs is to devise selection strategies that maximize information content with manageable resources. These strategies and methods can be deployed in the main stages of the breeding program: parental selection, early generation advancement and selection, and performance testing. With the emergence of phenomics tools and accompanying advances in statistical learning, primarily with machine learning, the prospects of more efficient integration of physiology based selection strategies on varying agro-ecological and agro-management systems have become feasible, and is a prime target for enhancing genomic prediction methods^52,71^. Fortuitously, these approaches provide an ability to do in-season SY prediction and cultivar management fit thereby somewhat alleviating the more resource intensive phenotypic characterization by machine harvesting of all potential cultivars creating opportunities for breeder to expand the size of the program without increasing costs leading to a higher genetic gain and operation efficiency. These strategies are conditional on finding the optimum suite of predictor traits that are agnostic to germplasm, infrastructure and breeder biases^72^. A realization of this multi-disciplinary effort can inform breeders on the genetic potential of their candidate cultivars to enable optimal selection and placement in the agro-management systems.

The knowledge generated from such an approach lends itself to develop prescriptive cultivars, that are developed for a specific agronomic production system and environment using breeding, genetics/genomics, phenomics, optimized breeding designs and selection strategies to combine the prescribed characteristics to meet the producer’s unique conditions. To accomplish this aim, high-throughput phenotyping (using ground and aerial imaging systems and different sensors) linked advanced computational tools such as ML, and genomic tools are required. Knowledge of the underlying physiological processes driving SY in contrasting agro-management systems is one of the first steps to implement prescriptive cultivar development strategy.

## Supplementary information

Supplementary Tables and Figures

## Data Availability

Data generated or analyzed during this study are included in this published article.
