# Supplementary material for: Machine Learning Approach for Prescriptive Plant Breeding
Source: Sci Rep. 2019 Nov 20;9:17132. doi: 10.1038/s41598-019-53451-4 (PMC6868245; doi:10.1038/s41598-019-53451-4)
Supplement: Supplementary file 1 — Supplementary Tables and Figures [file 41598_2019_53451_MOESM1_ESM.pdf]

Machine Learning Approach for Prescriptive Plant Breeding

Kyle A. Parmley[1], Race H. Higgins[1], Baskar Ganapathysubramanian[2], Soumik Sarkar[2], Asheesh K. Singh[1*]

Supplementary Table S1 Subset of SoyNAM genotypes evaluated across nine environments from 2014 to 2016.

Landrace (PI)	Diverse	High Yield (Elite)
PI 398881	LG94-1128	4J105-3-4
PI 404188A	LG00-3372	5M20-2-5-2
PI 427136	LG90-2550	CL0J95-4-6
PI 437169B	LG98-1605	CL0J173-6-8
PI 507681B	LG03-2979	HS6-3976
PI 518751	LG05-4832	LD01-5907
PI 561370	LG92-1255	LD02-4485
PI 574486	LG04-4717	Maverick
	LG97-7012	NE3001
	LG05-4464	Prohio
		Skylla
		U03-100612

Supplementary Table S2 GPS coordinates and observed environmental conditions of testing environments where 32 genotypes of the SoyNAM diversity panel were phenotyped and seed yield measured in contrasting agro-management systems.

				Environmental Conditions				
**Experiment**	**Year**	**Environment (County)**	**GPS Coordinates**	**Mean Min Temp (˚C)**	**Mean Max Temp (˚C)**	**Mean Relative Humidity (%)**	**Cumulative Solar Radiation (MJ)**	**Cumulative Precipitation (in)**
IA-RS	2015	1 (Boone)	42.018773, -93.771428	59	78	81	2910	29
		2 (Story)	42.011277, -93.733884	59	78	81	2910	29
	2016	3 (Boone)	42.009966, -93.788575	60	80	78	3051	25
		4 (Boone)	42.014145, -93.787124	60	80	78	3051	25
		5 (Cass)	41.330982, -95.183034	59	81	79	2852	26
IA-SD	2014	1 (Story)	41.998856, -93.696969	58	78	79	2877	26
	2015	2 (Story)	42.010934, -93.731847	59	78	81	2910	29
		3 (Boone)	42.013878, -93.787441	59	78	81	2910	29
		4 (Warren)	41.350007, -93.404313	59	80	83	2913	28

Note: Environmental conditions were collected and compiled from the Iowa State University Soil Moisture Network (https://mesonet.agron.iastate.edu/agclimate/#tmpf). Presented data are from May 1 – September 30 for each year and the nearest monitoring station used to the testing location.

Supplementary Table S3 Vegetative indices computed from hyperspectral reflectance wavelengths in the experiment (IA-RS and IA-SD).

Name	Index	Equation[a]	Original Source
Photochemical Reflectance Index	PRI	$(\rho_{531} - \rho_{570})/ (\rho_{531} + \rho_{570})$	Peñuelas et al., 1995
Ratio Analysis of Reflectance Spectra A	RARSa	(ρ_{675}/ρ_{700})	Chappelle et al., 1992
Ratio Analysis of Reflectance Spectra B	RARSb	$(\rho_{675}/ (\rho_{650} \text{ x } \rho_{700})$	Chappelle et al., 1992
Plant Senescence Reflectance Index	PSRI	$(\rho_{680} - \rho_{500})/ \rho_{750}$	Merzlyak et al.,1999
Vogelmann's Red Edge Index 2	VREI2	$(\rho_{734} - \rho_{747})/ (\rho_{715} + \rho_{726})$	Vogelmann et al., 1993
Normalized Difference Vegetation Index	NDVI	$(\rho_{780} - \rho_{670})/ (\rho_{780} + \rho_{670})$	Rouse, 1973
Renormalized Difference Vegetation Index	RDVI	$(\rho_{800} - \rho_{670})/ \text{Sqrt } (\rho_{800} - \rho_{670})$	Roujean and Breon, 1995
Normalized Multi-band Drought Index	NMDI	$(\rho_{860} - (\rho_{1640} - \rho_{2130}))/ (\rho_{860} + (\rho_{1640} + \rho_{2130}))$	Wang and Qu, 2007

[a] p is reflectance and the subscript is wavelength (nm).

Supplementary Table S4 ANOVA table for fixed effects in both experiments (IA-RS and IA-SD).

	IA-RS		IA-SD	
**Source of Variation**	**F value and significance level**	**df**	**F value and significance level**	**df**
Location (l)	119^{**}	4	273^{**}	3
Genotype (g)	7.4^{**}	31	23.2^{**}	31
Genotype x Location (gl)	1.8^{**}	124	1.8^{**}	93
Management Treatment (t)	8.0^{*}	1	36.9^{**}	2
Management x Genotype (gt)	1.1	31	<1	62

* Significant at the 0.05 level

** Significant at the 0.01 level

Supplementary Table S5 Descriptive statistics of seed yield (kg ha^{-1}) for agro-management systems experiments (IA-RS and IA-SD)**.**

		Seed Yield (kg ha^{-1})					
**Experiment**	**Treatment**	**N**	**Mean**	**Std. Dev**	**Min**	**Max**	**Repeatability (H^2)**
IA-RS	38	465	3203.6	595.8	1713.6	4883.5	0.95
	76	474	3146.8	570.7	1726.2	4927.4	0.96
IA-SD	Low	377	2886.6	800.1	878.8	4645.0	0.78
	Med	378	3226.9	772.5	1048.3	5235.0	0.78
	High	378	3215.8	801.9	1010.6	5542.6	0.81

Supplementary Table S6 Description of physiological traits included in random forest using ‘sizeTolerance’ function in ‘caret’ R package to identify informative subset of predictor variables.

**Combined**	**IA-RS**		**IA-SD**		
**All**	**38cm**	**76cm**	**Low**	**Med**	**High**
SPAD_S1	LAI_S2	SPAD_S3	CT_S3	SPAD_S3	CT_S3
SPAD_S2	SPAD_S1	VI_S1_RARSa	iPAR_S1	VI_S2_PRI	SPAD_S3
SPAD_S3	VI_S2_RARSa	VI_S2_VREI2	VI_S2_VREI2	VI_S3_PRI	VI_S2_PRI
VI_S2_VREI2	VI_S3_NMDI	VI_S3_NMDI	VI_S3_NDVI		VI_S2_VREI2
VI_S3_VREI2	VI_S3_PRI	VI_S3_VREI2	VI_S3_PRI		VI_S3_NDVI
VI_S3_NDVI	VI_S3_RARSb		VI_S3_PSRI		VI_S3_PRI
VI_S3_NMDI	VI_S3_VREI2		VI_S3_VREI2		VI_S3_VREI2
VI_S3_RARSb					

Supplementary Table S7 Results of recursive feature elimination random forest models trained using only a subset of the predictor traits that optimized model performance. Additional models were trained using a reduced subset using the ‘sizeTolerance’ function to further decrease the number of predictor variables included without increasing OOB RMSE more than 5% when compared to model with optimal performance.

			OOB Train Performance		Test Performance		Ranking Performance		
**Experiment**	**Treatment**	**# Features[a]**	**RMSE**	**R^2**	**R^2**	**RMSE**	**BACC**	**SEN**	**SPE**
Combined	All	30/8	299/319	0.68/0.63	0.63	244	0.79	0.67	0.92
IA-RS	38cm	25/7	324/346	0.58/0.53	0.44	243	0.77	0.63	0.91
	76cm	15/5	339/358	0.48/0.44	0.40	247	0.72	0.55	0.95
IA-SD	Low	13/7	232/241	0.82/0.80	0.80	205	0.88	0.81	0.95
	Med	25/3	313/331	0.69/0.64	0.66	253	0.77	0.63	0.91
	High	32/7	293/307	0.69/0.67	0.60	228	0.84	0.75	0.94

[a] Number of predictor variables included in model with; lowest RMSE value/ 5% range of lowest RMSE.

Supplementary Figures

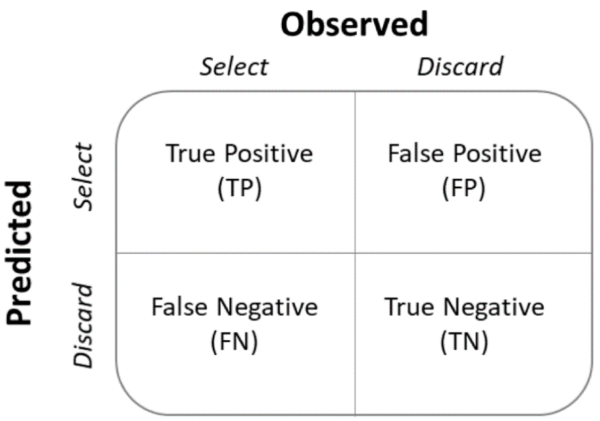

$$Balanced\ Accuracy\ (BACC) = \frac{SEN + SPE}{2}$$

$$Sensitivity\ (SEN) = \frac{TP}{TP + FN}$$

$$Specificty\ (SPE) = \frac{TN}{TN + FP}$$

Supplementary Figure S1 Confusion matrix and classification performance metrics to access RF classifier performance.

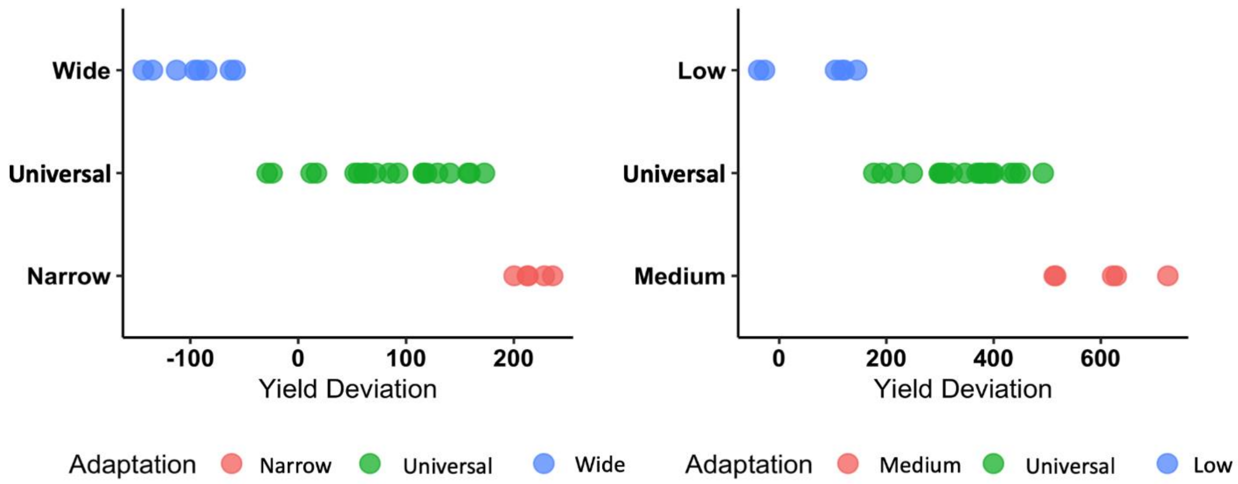

Supplementary Figure S2 Genotype adaptation classes assessed by computing yield deviation for IA-RS study (left) and IA-SD study (right) contrasting treatment levels.

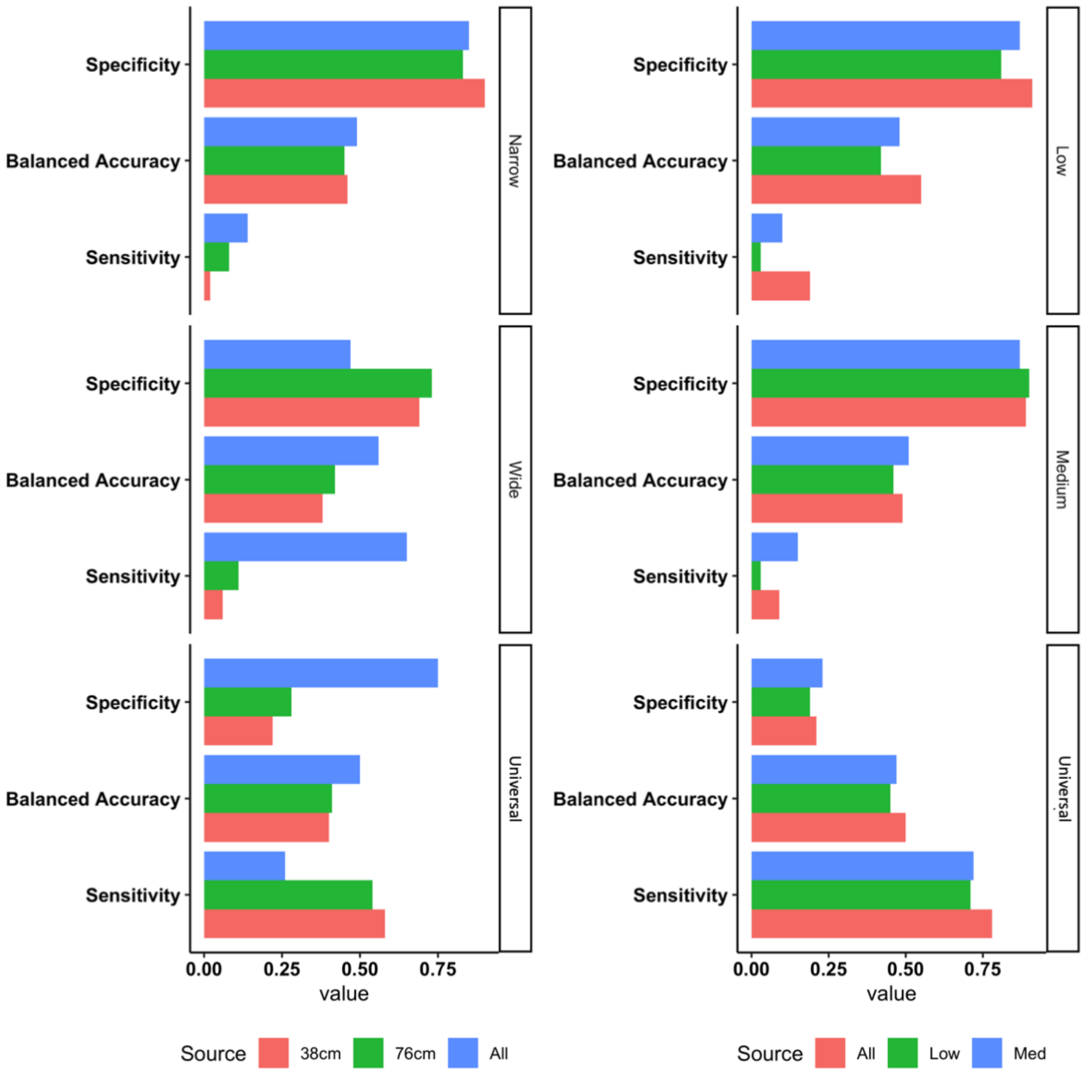

Supplementary Figure S3 Random Forest classifier performance of predicting genotype management fit conditional on the agro-management treatment levels from where training data were used.

List of abbreviations

**Abbreviation**	**Definition**
BACC	Balanced Accuracy
BLUP	Best Linear Unbiased Predictor
CT	Canopy Temperature
FN	False Negative
FP	False Positive
HTP	High Throughput Phenotyping
iPAR	Intercepted Photosynthetically Active Radiation
LAI	Leaf Area Index
ML	Machine Learning
MTA	Mean Tilt Angle
OOB	Out-of-bag error
PRE	Precision
R^2	Coefficient of determination
RF	Random Forest
RFE	Recursive Feature Elimination
RMSE	Root Mean Square Error
SEN	Sensitivity
SoyNAM	Soybean Nested Association Mapping
SPAD	Leaf chlorophyll content
SPE	Specificity
SY	Seed Yield
TN	True Negative
TP	True Positive
VI	Vegetative Indices
